# Traditional Societal Practices Can Avert Poor Dietary Habits and Reduce Obesity Risk in Preschool Children of Mothers with Low Socioeconomic Status and Unemployment

**DOI:** 10.3390/bs11040042

**Published:** 2021-03-24

**Authors:** Aleksandra S. Kristo, Angelos K. Sikalidis, Arzu Uzun

**Affiliations:** 1 Department of Food Science and Nutrition, California Polytechnic State University, San Luis Obispo, CA 93407, USA; asikalid@calpoly.edu; 2 Department of Nutrition and Dietetics, Istanbul Yeni Yuzyil University, Istanbul 34010, Turkey; arzu_._@hotmail.com

**Keywords:** health, lifespan nutrition, maternal child nutrition, nutritional habits, preschool children, socioeconomic status (SES)

## Abstract

Healthy nutritional habits are of vital importance for good health and quality of life for all individuals in all life stages. Nutritional habits shaped in early childhood set the foundation for future dietary practices applied through lifespan, hence informing risk towards chronic diseases. A key contributor to child health is maternal impact. A healthy childhood status translates into increased lifespan, health, and life-quality, as well as better family and social interactions and improved academic performance. These conditions can contribute to a healthier and more vibrant workforce, and thus extend positive impact on the economic and overall development of a country. Evidence related to maternal impact on childhood dietary habits is limited in Turkey, an emerging economy with notable disparities and a significant segment (approximately one third) of its 83 million population under the age of 30. Hence, the aim of this study was to investigate the relationship between the socioeconomic status (SES) of mothers on the dietary habits of their preschool children. A pilot cross-sectional observational study was conducted involving the mothers of 109 preschool children aged 4–6 years. Data on the nutritional status of children were collected through a food frequency questionnaire and a 24-h recall interview, while sociodemographic information was also collected, and statistical analyses conducted. An unexpected finding regarding the lack of association between the socioeconomic and employment status of mothers and the nutritional intakes of their children was observed. Interestingly, it appears that more traditional societal and nutritional practices typically undertaken in the case of lower SES, especially in more traditional settings like the case of Turkey, appear to extend a protective effect as per the nutritional habits and ensuing obesity risk in the case of children of women with lower SES. Another way to interpret our finding is that the difference in education and SES is not adequate to produce a significant effect in terms of children dietary intake. This is an interesting finding that warrants further study. Additionally, such studies serve to increase awareness about the importance of healthy nutritional practices in preschool children and the critical role of the mother on their nutritional status, particularly in the Turkish idiosyncratic setting.

## 1. Introduction

Nutritional habits shaped during early childhood lay the foundation for future dietary practices applied through lifespan, hence informing risk towards chronic diseases [1,2,3,4]. The role of family and particularly that of the mother is critical towards establishing habits and dietary patterns of children especially early-on and therefore should carefully be undertaken in a manner supportive towards the establishment of healthy nutritional practices for children [2,4]. Family awareness, knowledge, and attitude regarding nutrition and diet constitute key parameters, important for forming healthy eating practices in children [5]. Notably, nutritional habits do not only affect children’s physical growth and health but also their psychosocial and emotional development in addition to language and motor development [6,7,8]. Children mimic and finally adapt to the food choices, eating patterns and behaviours modelled by family members as they grow up [9]. Correcting suboptimal practices established during early childhood through positive behavioural modification is significantly demanding, inconvenient and challenging. A more effective approach is preventing children from establishing poor habits, by reinforcing a combination of healthy nutritional practices and proper education synergistically through family and school [6,10].

While both parents play an important role in the formation of healthy eating behaviours of their children, in most families, particularly those of a more traditional cultural background such as Turkey, mothers usually spend more time on food preparation and other activities related to family nutrition such as grocery shopping and meal planning [11,12,13]. The role of mothers regarding childcare is highly emphasized in most traditional cultures like the one seen in Turkey. Women raised in the typical Turkish cultural setting do usually have notable experience and receive training in this area. This is achieved by observing their own mother or other female relatives during meal preparations, by being involved as helpers as they grow up and by eventually adopting their mother’s systems, practices and certain routines. Furthermore, the adoption of certain beliefs around food, food purchases and preparation also contribute to eating behaviors. Independent of the factor “culture” (i.e., customs involving food/meal/diet related practices), in low- and middle-income countries, mothers are usually more involved in children nutrition, especially in the early years [11].

Socioeconomic factors such as income, employment and education are critical determinants for the dietary habits and nutritional status of children in developing countries [11,12,13,14,15,16,17,18,19,20,21,22,23,24,25]. Since mothers play an important role in shaping dietary habits of children, modifiers influencing mothers may indirectly extend over children in terms of dietary habits and health [23,25]. Research by Mondal et al. showed that maternal education is important for child health and development [26]. However, in a systematic review published in 2019, it was concluded that education of mothers is not necessarily a qualifier for better dietary habits [27]. It is postulated that specific nutrition/diet-related knowledge needs to equip the individual in order to have a significant impact on dietary habits. In another study, Emina et al. showed that maternal education was positively associated with children’s nutritional status with the children of educated mothers experiencing lower prevalence of malnutrition [28]. Moreover, in an observational study, Halder and Kejriwal showed that maternal nutritional education helps towards proper development in preschool children (3–4 years old). The work specifically demonstrated that child nutritional status and child growth improve as maternal nutritional awareness increases [29]. In children, healthier eating habits can support better academic performance [6,30]. Moreover, child health is shown as an important indicator of the lifespan and life quality, as well as the economic development of a country [6,30,31].

Turkey is considered an emerging economy and a country undergoing epidemiological and nutritional transition, similar to transitions seen in China and India. Westernization of the country and rapid urbanization increases parallel to the increase in fast food chains and consumption of packaged, highly processed, calorie-dense ready-to-eat foods and snacks [9]. There are also significant disparities observed in terms of education, employment and income within the population especially when considering women [32]. Simultaneously, large segments of the population in Turkey are characterized by traditional structures and practices. While a formal definition of the term “traditional structures and practices” does not exist, for the purposes of our study herein and in the context of the Turkish society, this term is approached as: large families with involvement of extended and elderly members in the daily routines, patriarchy, and mothers primarily occupied with household related chores and taking care of children.

The aim of this study was to investigate the relationship between socioeconomic and employment status of mothers on the dietary habits of their preschool children after appropriate grouping of participants. The three different variables constituting important determinants of SES studied were: maternal educational level, maternal income level and maternal employment status. We hypothesised (H_o_) that there is a positive correlation between maternal socioeconomic status and the quality of dietary intake of their children.

## 2. Materials and Methods

### 2.1. Study Design, Participating Population and Characteristics

**Figure 1:**
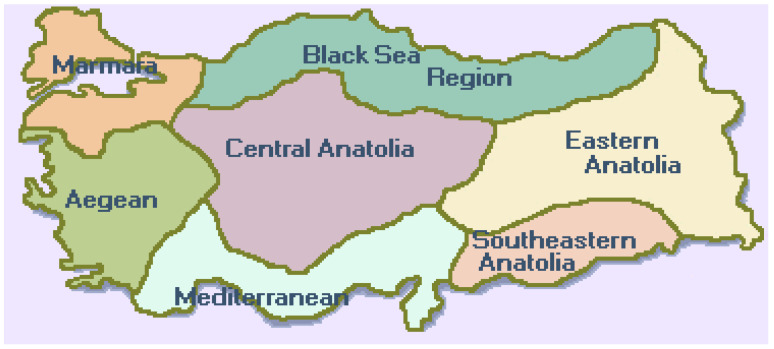
The seven geographical at-large regions of Turkey. The study was conducted in the city of Istanbul (Marmara region).

This was a cross-sectional pilot study involving the mothers of 188 preschool children aged 4–6 years. The study was performed at three different public preschool settings in Istanbul, Marmara region, Turkey (Figure 1), namely: Emir Sultan school and Behiye, Dr. Nevhiz Işıl school located in the Bahçelievler borough, and Penyelüks Hasan Gürel school located in the Küçükçekmece borough. All necessary meetings with the school principals and parents of students were performed prior to the commencement of the study. Participants were informed about the study prior to enrolment in the study and inclusion was solely on a strictly voluntary basis without incentives. All study participants signed an informed consent form prior to study commencement. Inclusion criteria were the following: participants were pre-menopausal, non-pregnant adult mothers without medical conditions (none diagnosed), able to work (no disability), married and not previously divorced with one child in preschool under full-time enrolment attending public preschool in Istanbul. Children were free of medical conditions (none diagnosed) and dietary restrictions (no food-related allergies/intolerances) and attended all preschool functions normally. There were initially 188 mothers meeting the inclusion criteria who volunteered and signed the informed consent form and enrolled in the study. Progressively there was a total dropout rate of 42%, thus leading to an actual study working population that consisted of 109 mothers and their 109 corresponding preschool children, one from each mother (N = 109). Participants were conveniently sampled to constitute a trial cohort for a pilot study employing a proof-of-concept approach. Children were aged 4–6 and were 66 female and 43 males. There is probably low interest and commitment in participating in such research studies as they are not conducted often in this population, hence it is not surprising to experience such dropout rates.

### 2.2. Methods and Data Collection

The outcome variables assessed were employment status, educational and income levels of the participating mothers. Each of the variables was further divided into two separate groups, such as employed-unemployed, high educational level-low educational level and high-income level vs. low-income level, in order to compare the nutritional intake of the children for each group. Grouping qualifiers for the outcome variables of educational level and income were used as per Turkish standards as reported previously [33]. More specifically, high educational level refers to higher level education (post-secondary education) whereas low pertains to up to secondary education level. Food frequency questionnaires (FFQ) on the nutritional intakes of preschool children for the past three months were completed by all participating mothers. A set of 12 additional follow-up questions regarding the dietary habits of children were included, aiming to better assess the nutritional status of children. Another set of namely socio-demographic questions was included in the questionnaire as a separate section as reported previously [33]. In addition, a 24-h recall of nutritional consumption regarding the children was performed during face-to-face interviews with the participating mothers. All interviews were conducted by the same trained Registered Dietitian Nutritionist. A validated FFQ previously used in a similar setting was applied after adding country-specific foods and modifications reflecting the Turkish food availability and culture [34]. The height and weight of children were reported by the mothers according to recent pediatrician visit. Body mass index (BMI) was calculated by dividing weight (kg) by height (m) squared (m^2^). Obesity was defined as a BMI index above the 95th percentile [35]. When evaluating the normal BMI values for girls and boys, the respective BMI-for-age growth charts published by WHO and reference values specific for Turkish children published by Neyzi et al., were used according to established methods [36]. Food frequency questionnaire and 24-h dietary recall results were analyzed by diet analysis software specially developed for Turkey: Beslenme Bilgi Sistemi (BeBiS) [37].

### 2.3. Statistical Analysis

Statistical analyses were performed using IBM SPSS version 24. Independent samples two-way ANOVA was performed on each group of variables for interaction identification. Two-way ANOVA was used to identify potential interactions between income and education level. None was found and as such those were not reported. Given no interactions were identified, follow-up t-tests were used to make the comparisons presented in the results section. Significance was accepted at *p* < 0.05.

### 2.4. Ethics Statement

All participant mothers gave their informed consent for inclusion upon their recruitment in the study. The study was conducted in accordance with the Declaration of Helsinki, and the protocol was approved by the Ethics Committee of Istanbul Yeni Yuzyil University (SBF-130705061-2017).

## 3. Results

### 3.1. Demographic Characteristics

Educational and income characteristics of participating mothers is shown on Table 1. Maternal employment, educational and income levels as related to energy, macronutrient and selected micronutrient intakes of their children, are presented on Table 2 and Table 3. The majority of participating mothers were unemployed (*n* = 81, 74.3%) versus employed mothers (*n* = 28, 25.7%). With regard to education and income, 45 (41.3%) mothers were of upper and 64 (58.7%) of low education levels, while 74 (67.9%) and 35 (32.1%) mothers were of middle- and low-income levels, respectively.

### 3.2. Maternal Employment Status

Based on our results, maternal employment, did not appear to extend any effect on the macronutrient intake of their preschool children. Both groups exhibited similar values in terms of macronutrient intakes and adequacy of nutrient intake for children. Interestingly there was still a marginally higher percentage of inadequately nurtured children energy-wise (daily caloric intake) in the children of the employed mothers, 66.7% versus 64.2% for the children of unemployed mothers. However, the majority of preschool children’s energy intake was fairly close to the requirements for their age group, yet marginally under the requirements, although not significantly.

The average energy intake of preschool children of unemployed mothers was 1373 kcal/d while children of employed mothers exhibited daily energy intake of 1303 kcal/d. The values of the two groups are of a similar order and there was no significant difference. In the group of the 28 working mothers, 11 (39.0%) children received an adequate amount of energy, while 17 (61.0%) did not quite meet the requirement set by the Turkish Ministry of Health for preschool children. In the group of 81 unemployed mothers, 29 (35.8%) of the preschool children received adequate amounts of calories, while 52 (64.2%) did not reach the minimum level set by the Turkish Ministry of Health. The average daily energy intake was 1321, 1398, and 1468 kcal for girls and 1410, 1492, and 1576 kcal for boys for the age groups 4, 5, and 6 years respectively (all mothers/children included), while the differences were not statistically significant.

Overall, no significant differences were found between the energy or macronutrient intakes of preschool children regardless of maternal level of education, income or employment status. Indicatively, the mean values for carbohydrate, protein and lipid daily intake levels in children of employed mother were: 151.1, 43.1, and 57.5 g/d respectively. The corresponding values for carbohydrate, protein and lipid daily intakes for the children of unemployed mothers were 156.9, 45.6, and 61.4 g/d respectively (Table 2). The results from the applied comparisons for the minerals, vitamins and fiber daily intake of children showed a similar trend to the results of those for energy and macronutrient intakes overall, for certain micronutrient intakes statistically significant differences were observed. More specifically, we did observe that children of unemployed mothers, as well as those of low education level, consumed a notably higher level of vitamin A/carotenoids and vitamin C. The same was seen as an effect of income level in favor of the children of mothers who were at the lower income level compared to middle income level (Table 3). The results for folic acid intakes also showed higher intakes for the children of mothers who were unemployed compared to employed, or in lower education or income level (Table 4). The statistically significant differences noted for vitamin A/carotenoids, vitamin C and folic acid, could be attributed to a slightly higher consumption of fruits, vegetables, and legumes in the children of the unemployed mothers.

Furthermore, there was no statistically significant difference between maternal employment and child BMI-for-age found in our study. The mean BMI-for-age values for the children of employed and not employed mothers were similar, namely 16.0 for the former and 16.3 for the latter group (Table 5).

### 3.3. Maternal Education Level

The highest education level attained by participating mothers was assessed via questionnaire. The results were the following: completed primary school education (*n* = 53, 46.6%), completed middle school education (*n* = 21, 19.3%), high school graduate (*n* = 27, 24.8%), university degree (*n* = 7, 6.4%) and completed postgraduate studies (*n* = 1, 0.9%) (Table 1). Completion of high school education was chosen as the borderline between high and low education among the mothers when dividing the educational level into two different groups to conduct the comparison of groups. The educational status of a mother was evaluated through classification in two different levels. High school education and lower (corresponding to low educational level) and greater than high school education (corresponding to high educational level). The number of participants with a completed high school education or higher was 35 (32.1%), while the group of lower than high school education comprised the majority of the participants reaching 74 (67.9%). In the cohort of the 35 mothers with a high school education or greater, 40.0% (*n* = 14) of children received an adequate number of daily calories while 60.0% (*n* = 21) of the children in the cohort had a nutritional intake technically below the recommended number of daily calories albeit not significant. Among the group with an education lower than high school (*n* = 74), 32.4% (*n* = 24) of the children received a sufficient daily calorie intake while 67.6% (*n* = 50) of children did not reach the minimum level of energy required for preschool children, according to the guidelines issued by the Turkish Ministry of Health. The majority of children with both higher and lower level of educated mothers, received an inadequate amount of energy. When comparing the two groups however, children of mothers with a lower education level receiving inadequate energy intake were more compared to the number of children of more educated mothers receiving insufficient caloric intake. However, most of the numbers were fairly close to the daily-recommended amounts of energy for preschool children as recommended by the Turkish Ministry of Health (less than 5% deviation), thus not rendering them significant in terms of their divergence. Hence, the gap between the amount of received energy and the recommended amount of energy intakes for children, was not nutritionally meaningful from a dietetics perspective.

The more educated group of mothers had children with a mean energy intake of 1328 kcal, while the children of lower educated women demonstrated a mean energy intake of 1377 kcal per day. There was no statistically significant difference between the educational status of the mothers and the energy intake of their children. The same observation applies to macronutrient intakes of children. The children of highly educated mothers exhibited an average intake of 151.4 g, 44.5 g, and 59.5 g of carbohydrate, protein and lipid respectively. The results from the lower educated mothers’ children were 158.2 g, 45.6 g, and 61.3 g for carbohydrate, protein, and lipid respectively. As observed, the nutritional intake of children (as in macronutrient intake) was independent of the educational status of their mothers. Taking our results together, we did not see a significant difference in BMI, energy and macronutrient intake in children regardless of the categorization of their mothers as per employment, education and income level status.

When comparing micronutrient intakes among children our results showed that children of non-employed mothers as well as those of lower education consumed a notably higher level of vitamin A/carotenoids and vitamin C. The same was seen as an effect of income level in favor of the children of mothers who were at the lower income bracket (Table 3). The results for folic acid intakes also showed higher intakes for the children of mothers who were non-employed, or in lower education or income level (Table 4). Maternal educational level had no effect on the consumption of vitamin B complex or other micronutrients’ intake assessed in children.

### 3.4. Maternal Income

Our results showed that there was no clear association between the income level of mothers and the nutritional habits of their preschool children. The income levels of the mothers were first divided into three different groups as low, middle and high income. The minimum wage of 1300 TL/month net (approx. 450 US dollars) was set as the lowest limit of income and was evaluated as low income. Middle income corresponded to net monthly earning between 1300 and 3500 TL (450—1220 US dollars) while high income was set as an income range of 3500–5000 TL (1220—1730 US dollars), however no participant reported income higher than 4000 TL (1385 US dollars) (Table 1). Brackets followed Turkish Ministry of Economy and Finances tables [33]. The number of mothers belonging to the three different income classification groups was as follows: low income (*n* = 35), middle income (*n* = 63) and high income (*n* = 11). The criteria for low income were stable, but middle and high incomes was gathered into one group as middle income because of very low numbers in the high-income category. Finally, 35 of the 109 participating mothers constituted the group of low-income mothers while 74 represented the group of middle income. No statistically significant results between the energy, macronutrients, vitamins, minerals, or fibre and maternal income were seen. We did not detect a maternal income effect on the nutritional intakes of their children. Child BMIs were also compared as per the income levels and no association was seen either (Table 5).

## 4. Discussion

In summary, there were no statistically significant differences in the results regarding the working status, educational level, or the income level of the mothers and the macronutrient nutritional intake or energy intake of their children. Moreover, there was no statistically significant difference in terms of children BMI relative to mothers’ status as per employment, income and education. We did observe, however, a higher intake for folic acid, vitamins A/carotenoids and C consistently for the children of mothers in the non-employed, low income, low education level, a counterintuitive finding, which may be partially explained by the provision of high-quality inexpensive produce (e.g.,: fruits, vegetables, meat and dairy) from the rural areas the urban families are connected to.

### 4.1. Maternal Employment Status and Time as Relating to Diet, Obesity and BMI of Children

Results from the Turkish Statistics Institute showing a low percent of working females especially compared to that of males in Turkey [38], was reflected in the results of our study as well. This can be explained in part by the labor inequality that is typically seen in Turkey and other countries in the Orient (e.g., Iran, Pakistan, India, Syria) [39,40,41]. Furthermore, in terms of our study’s participants, the financial and educational difference among the populations in Istanbul, fluctuates greatly depending on the district/borough in discussion. The results obtained from our work partly reflect the situation of the two districts Yenibosna and Sefaköy, which are considered typical working-class areas by Turkish standards. Research conducted by Cawley and Liu shows that maternal employment is associated with less time per day spent by mothers on activities associated with child diet/nutrition, such as grocery shopping, cooking and eating with children [42]. This condition arguably promotes a situation that enhances risk for obesity as there is less control over child dietary intakes, which can lead to unbalanced diets and an excess of caloric intake due to indulging dietary behavior, thus promoting obesity.

In our study however, there was no statistically significant difference between the energy intake of employed and unemployed mothers’ preschool children. Nevertheless, children of non-working mothers still exhibited a marginally greater daily caloric intake when compared to children of working mothers. Furthermore, there is no evidence that children of employed mothers demonstrate poor quality nutrition when compared with the unemployed mothers’ children. This is an interesting finding, potentially explained by the cultural idiosyncrasies, including but not limited to more traditional structures, involving extended family members and support systems. Living in a relatively more traditional and conservative society, Turks tend to place significant importance on cultural and family ties and values. A research/survey about family values in Turkey performed by the Turkish Prime Ministry General Directorate of Family and Social Research in Ankara, reports that Turkish families highly appreciate their elderly family members also when it comes to raising their children [43]. Elderly individuals are generally highly respected, and a large number of families are in close relationship with their older relatives. It is not uncommon that they are living close to each other, or even in the same household. One of the questions directed to the families was if they agreed with the statement that children are raised better in an environment together with the older family members such as the grandparents. Interestingly, 77.8% of the participating families agreed with the statement, while only 10% disagreed and 12.2% chose “unsure” as their answer. Consequently, there is recent evidence to suggest that parents in Turkey think that the elderly family members play an important role in the education and socializing of their children [39]. In this context, even if employed mothers share less time with their children, they likely receive help from grandparents or other relatives living closely. Unexperienced mothers engage in the practice of receiving advice, practical help and support in general from their experienced older family members.

Another reason explaining the lack of difference between nutrition of employed and unemployed mothers’ children, could be that most families originally come from rural areas in the country. Typically, some family members or other relatives live in the original locations in the rural areas, so even if the families participating in our work reside in the city, they are still in contact with their village. Organic farming is more common in those rural areas, especially small-scale for personal use, so there is a supply line for organic healthy food (e.g., fruits, vegetables, eggs, dairy, and meat) for the family residing in the city. Some families do even have their own garden, or have access to that of their relatives, thus making it easier to stock organic foods in certain periods. A majority of families in Turkey therefore have a stock of canned, dried or deep-frozen foods easy to prepare without having to do grocery shopping for various food items. Employed mothers typically spend less time with their children, but they also seek ways to increase their time and particularly the quality of time spent with their children. Some choose to work part-time or taking a break from their current workplace for some months or years when their child is still young. Even if mothers’ labor force participation has increased, working mothers still apparently try to protect their time-investment in children [43].

There is evidence supporting that children of working mothers consume more meals and snacks away from home, resulting in a higher overall caloric intake thus contributing to the development of obesity [44,45]. Food consumed away from home is typically more energy-dense and of poorer nutritional value particularly in relation to child needs for health, growth and development. Additionally, working mothers have less time for preparing meals and decreased opportunities to monitor their child’s meals, with both factors being potential contributors to childhood obesity as well.

According to our results, working status of the mothers produced no significant differences regarding the nutritional status of children. One potential reason for this finding might be that employed mothers still have the ability to monitor their children’s daily life via the caretaker which usually is the grandparents. Most of the times in the typical Turkish family setting, there is someone available to prepare home-cooked food and someone to encourage physical activity for children (e.g., walks and/or games at the local park) [46]. Research by Oddo et al., also demonstrated that in low-and middle-income countries children are likely to maintain their normal weight and healthy nutritional status independently of the employment status of their mother [47]. In the case of pre-schoolers, this could be attributed to the disciplined eating routines and structure commonly applied in kindergartens. Children grow accustomed to eating with regularity at the same time each day and the eating habits are incorporated into the overall daily routine. Eating together with their friends during fixed mealtimes every day makes the entire eating process more exciting and fun and helps children in establishing healthy eating patterns. A list containing fixed food groups for each day prepared by preschool teachers is used in the public preschool system in Turkey. These lists are used to provide healthy nutritive foods from various food groups, ensuring the variability in consumption by the child and minimizing tendencies towards other foods that the classmate might have that day. This way meals become more standardized and normalized and behavioral uniformity appears positive in terms of promoting the establishment of healthy eating habits. Mothers comply with this list and prepare a dish from the listed food group specific for each day.

Our results showed no statistically significant relationship between maternal employment and child BMI. A study conducted by Anderson et al., shows that children are more likely to be overweight if their mother works more intensively (more hours per week) [48]. This result applies to children of mothers with higher education and a high-income level. However, no evidence was found that the number of weeks a mother works over her child’s life predicts overweight development in the child. These observations suggest that the link between maternal employment and the child’s weight status might be the limitations of time faced by intensively working mothers. Working fewer hours per week gives the mother more time for shopping, cooking and activities for the child [48].

Further to the point of work intensity, Hawkins et al., showed that the probability of children being overweight increased with the number of hours that their mother worked per week. Interestingly however, this relationship was only significant for children from high-income families. More specifically, it was shown that long hours of maternal employment had a negative effect on three-year-old children living in the United Kingdom. This was the case if the mother held any employment since childbirth [49]. Non-standard work hours (working nightshifts, weekends or an irregular shift pattern) could also contribute to irregular patterns of child weight or BMI. Similar to Hawking’s group’s findings, Morrissey and colleagues found that an increase in the total time of maternal employment is associated with an increase in her child’s BMI [50]. Yet another study by Ziol-Guest et al. shows that the number of maternal work hours during the child’s lifespan is associated with higher BMI and overweight risk at ages 13 or 14, while these associations are more apparent among children of highly educated mothers. The authors postulate that the relationship between maternal employment and children’s BMI for the high-educated mothers could be sought into the role of screen time [51]. Although this is a plausible suggestion, it currently lacks consensus as per the degree to which it constitutes a cause or enough of an explanation regarding the phenomenon discussed.

On the flip side, there are studies reporting results which do not corroborate the notion that maternal employment contributes to higher BMI for children. Work performed by Taylor et al., found that full-time employment status of mothers was not associated with their child’s BMI [52]. Moreover, Speirs et al. concluded that amount of sleep and no other daily routines, or mother employment status, appeared to increase risk for higher BMI in children [53]. These results are in agreement with our findings presented herein. In summary, there is apparently no consensus regarding the effect of maternal employment and child BMI in the literature. In our study, we also compared the BMIs of children taking into consideration the differences in the level of income of their mothers, without finding an association between the two either. A possible explanation might be that no family received a lower income than the minimum wage. Another reason could be that even if a family has a shortage of income, food supplies are usually the last to be compromised. Families in Turkey will still obtain an adequate amount of essential nutrients, or they might use alternative cooking styles and preparation methods such as mixing the grain and legume group to produce high quality protein meals. Another commonly seen feature in Turkey is that a significant number of families bring foods from their villages, typically in the countryside where farming is common. These food items are typically less expensive and not registered in the economy’s cycle. This way, through the part of the family still living in the countryside, urban families are typically supplied with home-made products that are added to the food basket of the household.

### 4.2. Maternal Educational/SES Level and Child Nutrition

Similar to the lack of differences in the BMI and/or energy intake, the results from our study showed that nutrient intake of children was independent of the educational status of their mothers. More specifically, our results showed that energy, macronutrient, and fiber intakes of children were totally independent of both the employment and educational status of their mothers. Furthermore, with the exception of vitamins A (including carotenoids), C and folic acid there were no difference in the micronutrients either. These observations allude to the fact that there are other factors affecting nutritional intake of children. The educational system of Turkey as well as the general nutrition knowledge of mothers could offer a potential explanation of our findings. Dietitians and other health professionals are important sources of accurate information regarding nutritional practices applied for the general population, especially children. Television and social media can also be a beneficial medium when it comes to disseminating information to the public, provided appropriate regulation as per the scientific accuracy of information delivered is implemented for respective programs. Mothers are usually the most interested party towards heath and diet related information that is beneficial for their children’s growth and development and their mentality is found to affect children’s diet [54]. Independent of their education level, mothers interested in personal development are typically more open, interested and receptive to new information regarding their child’s healthy development which translates into better dietary choices [55]. This could be one reason explaining the similarity between the groups of higher and lower educated mothers. The education level of mothers does not appear to extend a significant effect on the nutritional habits of their children in our study. One would probably expect that mothers of higher education are more conscious about their child’s nutrition. However, given the fact that no specific nutrition education is provided through the formalized education system, nutrition knowledge acquisition mostly depends on the willingness of an individual (in this case the mother) to investigate, find and acquire valid information. Many television programs targeting mothers are available on Turkish television [56]. Other seminars and conferences arranged by schools and food catering companies towards the education of children, teachers, and families by nutritionists are other solutions that are becoming increasingly more available especially in the Istanbul area. In this context, there are increasingly more opportunities for mothers to obtain helpful information regarding maternal-child nutrition, at least on a practical level.

Other factors contributing to the results obtained could be of cultural nature, involving the food culture, which is very important in Turkish families, and the food supply chain. Turkish cuisine is largely influenced by both Mediterranean and Ottoman cuisines. Each of the seven at-large geographical regions in Turkey (Marmara, Aegean, Mediterranean, Central Anatolia, South-eastern Anatolia, Eastern Anatolia, and the Black Sea region; see Supplementary Figure S1) portrays its own style and specialities prepared with ingredients specific for that area. Children mostly learn how to cook by their mothers early during adolescence and the culture of eating regularly outside of home is not that common. Mothers have the opportunity to attend educational seminars held by an educational nurse during pregnancy. They are also followed-up by this nurse if needed after birth. Hence, mothers start receiving helpful information and practical guidance about nutrition during pregnancy. This way, the mother obtains useful information about early nutritional practices regarding her child and develops the habit of receiving information that can be incorporated into her life. As the child grows, regular controls at the family doctor or a child growth specialist helps the family to monitor the growth and development of their child [57]. In conclusion, Turkish families seem to pay extra attention towards the nourishment of their child and mothers are very concerned about their child’s nutrition, independent of their educational status. Furthermore, as many urban families typically maintain family ties with rural areas, they also receive produce and other foods grown organically for personal use by their extended family members. This is a possible explanation as per our finding on the higher intake of vitamin A (including carotenoids), vitamin C and folic acid in the arguably lower SES group. Additionally, an interesting study performed by Burchi F., showed that a high educational level attained by the mother would most likely play a minimal role in her child’s nutrition, by expanding her economic opportunities [58]. Other work conducted by Yabanci et al., in Turkey, showed that mothers specifically with higher nutrition knowledge were more likely to have children with normal weight [59]. Interventions aiming to improve education, health beliefs as well as empowerment through employment and better income for mothers have been shown to improve nutrition and health outcomes for young children primarily in developing countries [60].

In Europe, several studies have indicated the importance of family in terms of food choices as well as the diet, more so as a pattern and lifestyle practices, and how those factors influence the nutritive quality of diet and childhood obesity risk [61]. More specifically, a study comprised of 1728 primary school children in Greece, demonstrated that adherence to the Mediterranean Diet as a lifestyle plays a protective role against childhood overweight/obesity, particularly among children living in nuclear families [62]. Moreover, findings from the Healthy Lifestyle in Europe by Nutrition in Adolescence (HELENA) study in Europe, underlined the role of social engagement and encouragement of relatives and peers in adolescents’ diet quality. The work concluded that intervention or promotion programs aimed at enhancing diet quality in adolescents should target both family and peers [63].

Our study presented herein was conducted in Turkey. Turkey is interesting and idiosyncratic in the sense that it combines cultural, societal and financial structures that often times are perceived as an amalgamation of the Orient and Occident, as well as a mixture of progressive and conservative approaches. In our study, maternal education implies standard normal education received by every individual in Turkey and not necessarily specifically in nutrition. Nutrition education received from other sources such as newspapers, books, journals, informative educational television programs, and experienced family members such as parents and grandparents are quite common in the Turkish culture, independent of the standard educational status of a mother [64]. Basic nutrition education can arguably influence the quality of food provided to children. The understanding of the reason behind a suggested action and/or practice, helps mothers to take it more seriously, knowing that a healthy lifestyle later in life is formed during the early preschool years of a child. In a European report comparing the Turkish educational system to European ones regarding activities towards nutritional education programs, concluded that this type of education was insufficient in Turkey. No specific classes about nutrition exist in the curriculum of primary and secondary education [65,66,67]. Currently, new steps towards educational programs pertinent to nutrition, diet, food, and health are being taken. New regulations about the hygienic inspection of educational institutions’ food management and foods to be sold in school canteens and other school health activities, are being taken by the Ministry of National Education. A healthy nutrition and active lifestyle program have been issued by the Prime Ministry’s General Directorate of Personnel and Principles, aiming in the prevention of obesity and other chronic illnesses in 2010. Education of school canteen managers, school canteen inspections and a regulation towards diabetic students was also released by the Turkish Ministry of education. The Public Health Institution of The Turkish Ministry of Health published the Healthy Nutrition and an Active Lifestyle Program in Turkey. The protocol of this so-termed “diet-friendly” school program was started by the Turkish Ministry of National Education and the Ministry of Health together in 2010 but assessment and results are not yet conclusive [66]. All of these projects support an overall effort that can play an important role in promoting a healthy and balanced lifestyle for students. In short, the fact that the urban families are connected to their rural relatives for a supply of good quality food at low cost, in addition to help from family members in the raising of children as well as practical knowledge on food preparation passed from generation to generation all contribute positively towards improving the nutritional outcome of children. The key message of our exploratory pilot work here is that there is no significant difference in terms of quality of nutrient intake for children of mothers of different SES, apparently associated with traditional practices and norms typical in the Turkish society of working class.

Strengths of the present study include the understudied population and the realistic environment. A limited participant sample and mode of selection constitute potential weaknesses of the study herein.

## 5. Conclusions

Maternal-child nutrition and the influence of mothers on child dietary habits and overall development are all crucial to the proper development of a child. In a traditional and idiosyncratic setting like that of Turkey, it is interesting to investigate the association between maternal employment status and subsequent SES and nutritional intakes of children especially at the young preschool age. A number of previous studies either showed a positive relation between the employment status, educational or income level of the mother and the nutritional intakes of their child or no association whatsoever, hence making the derivation of a conclusion challenging, however illustrating the complexity of the matter and the fact that the answer is probably dependent on the particular conditions of the setting in which research is conducted. In that sense and given that the question is understudied in Turkey, our work was performed in an effort to address this question. In a preliminary pilot study with limited sample, we found that the intake of nutrients and energy in preschool children aged 3–6 in Turkey in two working-class areas of Istanbul was not affected by the working status and/or the SES of their mothers. We attributed these findings to idiosyncratic cultural characteristics reflecting a more traditional and somewhat conservative family-centered society typical of Turkey particularly in the working-class of today. In this context, we conclude that those structures may under certain circumstances extend a protective effect against adverse outcomes often times seen in conditions of poverty and/or low SES. Performing similar studies at other locations in Istanbul or even other cities will offer a wider perspective of the situation in Turkey, and potentially other countries under epidemiological and nutritional transition.

## Figures and Tables

**Table 1:** Education and Income characteristics of participating mothers.

**Highest Education Level Attained**	**N (%)**
Primary-school	53 (48.7)
Middle-school	21 (19.3)
High-school	27 (24.7)
University (BSc)	7 (6.4)
University (post-graduate)	1 (0.9)
**Income**	**N (%)**
High (>3500 TL/month net; approx. $1220)	11 (10.1)
Medium (1300–3500 TL/month net; approx. $450—$1220)	63 (57.8)
Low (<1300 TL/month net; approx. $450)	35 (32.1)
TL: Turkish Lira/$: US dollars	

**Table 2:** Maternal employment status, education and income levels of mothers vs. energy and macronutrient (Carbohydrate, Protein and Lipid) intake of their preschool children.

	**Employment Status**	**N (%)**	**Mean** ** ± SEM**
Energy (kcal/day)	Employed	28 (25.7)	1302.6 ± 108.1
	Non-employed	81 (74.3)	1372.4 ± 68.6
CHO (g/day)	Employed	28 (25.7)	151.1 ± 14.4
	Non-employed	81 (74.3)	156.9 ± 7.2
Protein (g/day)	Employed	28 (25.7)	43.1 ± 4.6
	Non-employed	81 (74.3)	45.6 ± 2.2
Lipid (g/day)	Employed	28 (25.3)	57.5 ± 6.6
	Non-employed	81 (74.3)	61.4 ± 4.4
	**Education Level**	**N (%)**	**Mean** ** ± SEM**
Energy (kcal/day)	Upper	45 (41.3)	1327.7 ± 95.9
	Low	64 (58.7)	1377.2 ± 75.7
CHO (g/day)	Upper	45 (41.3)	151.5 ± 12.1
	Low	64 (58.7)	158.2 ± 7.5
Protein (g/day)	Upper	45 (41.3)	44.6 ± 3.4
	Low	64 (58.7)	45.6 ± 2.4
Lipid (g/day)	Upper	45 (41.3)	59.5 ± 5.3
	Low	64 (58.7)	61.3 ± 5.2
	**Income Level**	**N (%)**	**Mean** ** ± SEM**
Energy (kcal/day)	Low	35 (32.1)	1412.0 ± 162.7
	Middle	74 (67.9)	1342.0 ± 57.9
CHO (g/day)	Low	35 (32.1)	156.5 ± 13.8
	Middle	74 (67.9)	155.6 ± 7.3
Protein (g/day)	Low	35 (32.1)	49.1 ± 4.8
	Middle	74 (67.9)	44.2 ± 2.1
Lipid (g/day)	Low	35 (32.1)	64.3 ± 11.5
	Middle	74 (67.9)	59.4 ± 3.3

CHO: Carbohydrate intake; SEM: Standard Error of Mean; Reference dietary values are provided in the Supplement (Tables S1–S4).

**Table 3:** Association of maternal employment status, education and income levels and dietary intake of minerals, vitamins and fiber of children.

	**Employment Status**	**N (%)**	**Mean** ** ± SEM**
Ca (mg/day)	Employed	28 (25.7)	614.4 ± 64.8
	Non-employed	81 (74.3)	724.5 ± 40.3
Fe (mg/day)	Employed	28 (25.7)	5.75 ± 0.6
	Non-employed	81 (74.3)	6.9 ± 0.4
**Vit A/Carotenoids** (IU/day)	Employed	28 (25.7)	491.2 ± 67.9
	Non-employed	81 (74.3)	**739.3 ± 90.9** *
Vit D (IU/day)	Employed	28 (25.7)	1.2 ± 0.3
	Non-employed	81 (74.3)	3.1 ± 0.9
Vit E (mg/day)	Employed	28 (25.7)	11.1 ± 1.4
	Non-employed	81 (74.3)	13.7 ± 1.5
Vit C (mg/day)	Employed	28 (25.7)	66.6 ± 8.6
	Non-employed	81 (74.3)	**90.3± 7.8** *
Fiber (g/day)	Employed	28 (25.7)	11.1 ± 1.1
	Non-employed	81 (74.3)	15.1 ± 1.0
	**Education Level**	**N (%)**	**Mean** ** ± SEM**
Ca (mg/day)	Upper	45 (41.3)	660.2 ± 51.7
	Low	64 (58.7)	728.7 ± 46.4
Fe (mg/day)	Upper	45 (41.3)	6.35 ± 0.51
	Low	64 (58.7)	6.94 ± 0.44
**Vit A/Carotenoids** (IU/day)	Upper	45 (41.3)	536.54 ± 64.8
	Low	64 (58.7)	**780.4± 110.9** *
Vit D (IU/day)	Upper	45 (41.3)	3.8 ± 1.7
	Low	64 (58.7)	2.1 ± 0.5
Vit E (mg/day)	Upper	45 (41.3)	11.9 ± 1.3
	Low	64 (58.7)	13.9±1.9
**Vit C** (mg/day)	Upper	45 (41.3)	90.1 ± 8.6
	Low	64 (58.7)	**129.9± 55.4** *
Fiber (g/day)	Upper	45 (41.3)	13.1 ± 1.3
	Low	64 (58.7)	14.9 ± 1.1
	**Income Level**	**N (%)**	**Mean** ** ± SEM**
Ca (mg/day)	Low	35 (32.1)	718.2 ± 68.4
	Middle	74 (67.9)	700.8 ± 41.2
Fe (mg/day)	Low	35 (32.1)	7.5 ± 1.0
	Middle	74 (67.9)	6.5 ± 0.3
**Vit A/Carotenoids** (IU/day)	Low	35 (32.1)	**894.8± 56.6** *
	Middle	74 (67.9)	626.4 ± 52.7
Vit D (IU/day)	Low	35 (32.1)	4.4 ± 1.1
	Middle	74 (67.9)	2.2 ± 0.5
Vit E (mg/day)	Low	35 (32.1)	16.1 ± 0.4
	Middle	74 (67.9)	12.3 ± 0.9
**Vit C** (mg/day)	Low	35 (32.1)	106.8 ± 16.5
	Middle	74 (67.9)	**96.6± 15.1** *
Fiber (g/day)	Low	35 (32.1)	16.6 ± 0.5
	Middle	74 (67.9)	13.4 ± 0.8

An asterisk (*) and bold denote statistically significant difference at *p* < 0.05. All other comparisons yielded *p* > 0.1 values; Nutrients whereby a statistically significant difference was found are shown in bold. ANOVA analyses results reflected in the table. SEM: Standard Error of Mean. Reference dietary values are provided in the Supplement (Tables S1–S4).

**Table 4:** Association of maternal employment status, education, income levels and dietary intake of B-complex vitamins for children.

	**Employment Status**	**N (%)**	**Mean** ** ± SEM**
Thiamin (B_1_) (mg/day)	Employed	28 (25.7)	0.55 ± 0.04
	Not employed	81 (74.3)	0.60 ± 0.1
Riboflavin (B_2_) (mg/day)	Employed	28 (25.7)	0.95 ± 0.1
	Not employed	81 (74.3)	1.10 ± 0.06
Niacin (B_3_) (mg/day)	Employed	28 (25.7)	5.89 ± 1.14
	Not employed	81 (74.3)	6.71 ± 0.62
Pantothenic Acid (B_5_) (mg/day)	Employed	28 (25.7)	2.81 ± 0.29
	Not employed	81 (74.3)	3.40 ± 0.19
Pyridoxine (B_6_) (mg/day)	Employed	28 (25.7)	0.92 ± 0.15
	Not employed	81 (74.3)	1.04 ± 0.09
Biotin (B_7_) (μg/day)	Employed	28 (25.7)	23.10 ± 2.68
	Not employed	81 (74.3)	29.35 ± 2.36
Vit B_12_ (μg/day)	Employed	28 (25.7)	3.08 ± 0.51
	Not employed	81 (74.3)	3.44 ± 0.28
**Folic Acid total** (μg/day)	Employed	28 (25.7)	125.71 ± 13.10
	Not employed	81 (74.3)	**168.36± 10.20** *
	**Education Level**	**N (%)**	**Mean** ** ± SEM**
Thiamin (B_1_) (mg/day)	Upper	45 (41.3)	0.54 ± 0.05
	Low	64 (58.7)	0.62 ± 0.04
Riboflavin (B_2_) (mg/day)	Upper	45 (41.3)	0.98 ± 0.08
	Low	64 (58.7)	1.10 ± 0.06
Niacin (B_3_) (mg/day)	Upper	45 (41.3)	6.62 ± 0.85
	Low	64 (58.7)	6.54 ± 0.71
Pantothenic Acid (B_5_) (mg/day)	Upper	45 (41.3)	3.24 ± 0.26
	Low	64 (58.7)	3.44 ± 0.21
Pyridoxine (B_6_) (mg/day)	Upper	45 (41.3)	0.96 ± 0.13
	Low	64 (58.7)	1.05 ± 0.08
Biotin (B_7_) (μg/day)	Upper	45 (41.3)	25.96 ± 1.85
	Low	64 (58.7)	29.97 ± 2.91
Vit B_12_ (μg/day)	Upper	45 (41.3)	3.42 ± 0.51
	Low	64 (58.7)	3.35 ± 0.26
**Folic Acid total** (μg/day)	Upper	45 (41.3)	145.81 ± 12.34
	Low	64 (58.7)	**168.71± 11.88** *
	**Income Level**	**N (%)**	**Mean** ** ± SEM**
Thiamin (B_1_) (mg/day)	Low	35 (32.1)	0.7 ± 0.1
	Middle	74 (67.9)	0.6 ± 0.03
Riboflavin (B_2_) (mg/day)	Low	35 (32.1)	1.1 ± 0.09
	Middle	74 (67.9)	1.1 ± 0.06
Niacin (B_3_) (mg/day)	Low	35 (32.1)	8.1 ± 1.6
	Middle	74 (67.9)	6.1 ± 0.5
Pantothenic Acid (B_5_) (mg/day)	Low	35 (32.1)	3.7 ± 0.4
	Middle	74 (67.9)	3.2 ± 0.2
Pyridoxine (B_6_) (mg/day)	Low	35 (32.1)	1.1 ± 0.14
	Middle	74 (67.9)	1.0 ± 0.1
Biotin (B_7_) (μg/day)	Low	35 (32.1)	33.2 ± 6.3
	Middle	74 (67.9)	26.5 ± 1.6
Vit B_12_ (μg/day)	Low	35 (32.1)	3.8 ± 0.7
	Middle	74 (67.9)	3.3 ± 0.2
**Folic Acid total** (μg/day)	Low	35 (32.1)	182.9 ± 4.2
	Middle	74 (67.9)	**153.1± 8.3** *

An asterisk (*) and bold denote statistically significant difference at *p* < 0.05, all other comparisons yielded *p* > 0.1 values. Nutrients whereby a statistically significant difference was found are shown in bold. ANOVA analyses results reflected in the table. SEM: Standard Error of Mean. Reference dietary values are provided in the Supplement (Tables S1–S4).

**Table 5:** Association of maternal, employment status, educational level and income level and BMI for age and sex of children.

	**Working Status**	**N (%)**	**Mean** ** ± SEM**
**BMI**	Employed	28 (25.7)	16.03 ± 0.72
	Not employed	81 (74.3)	16.32 ± 0.33
	**Education Level**	**N (%)**	**Mean** ** ± SEM**
**BMI**	Upper	45 (41.3)	16.45 ± 0.42
	Low	64 (58.7)	16.17 ± 0.40
	**Income Level**	**N (%)**	**Mean** ** ± SEM**
**BMI**	Low	35 (32.1)	16.75 ± 0.75
	Middle	74 (67.9)	16.11 ± 0.30

BMI: Body Mass Index (for age and sex as per WHO standards). SEM: Standard Error of Mean. All comparisons yielded *p* > 0.1 values.

## Data Availability

The data presented in this study are available upon request from the corresponding author. The data are not publicly available due to the potential classification as personal health data.

## Supplementary Materials

The following are available online at https://www.mdpi.com/2076-328X/11/4/42/s1, Table S1: Energy reference values for girls according to their physical activity levels, Table S2: Energy reference values for boys according to their physical activity levels, Table S3: Dietary reference values for protein intake, Table S4: Reference values for vitamin intake for both sexes age 4–6 years.

## References

[1] Scaglioni S., De Cosmi V., Ciappolino V., Parazzini F., Brambilla P., Agostoni C. (2018). Factors Influencing Children’s Eating Behaviours. Nutrients.

[2] Corkins M.R., Daniels S.R., de Ferranti S.D., Golden N.H., Kim J.H., Magge S.N., Schwarzenberg S.J. (2016). Nutrition in Children and Adolescents. Med. Clin. N. Am..

[3] Chaffee B.W., Feldens C.A., Rodrigues P.H., Vítolo M.R. (2015). Feeding practices in infancy associated with caries incidence in early childhood. Community Dent Oral Epidemiol..

[4] Kersting M., Alexy U., Schürmann S. (2016). Critical Dietary Habits in Early Childhood: Principles and Practice. World Rev. Nutr. Diet..

[5] Romanos-Nanclares A., Zazpe I., Santiago S., Marín L., Rico-Campà A., Martín-Calvo N. (2018). Influence of Parental Healthy-Eating Attitudes and Nutritional Knowledge on Nutritional Adequacy and Diet Quality among Preschoolers: The SENDO Project. Nutrients.

[6] Kristo A.S., Gültekin B., Öztağ M., Sikalidis A.K. (2020). The Effect of Eating Habits’ Quality on Scholastic Performance in Turkish Adolescents. Behav. Sci. (Basel).

[7] Black M.M. (2018). Impact of Nutrition on Growth, Brain, and Cognition. Recent Res. Nutr. Growth.

[8] Muhoozi G.K.M., Atukunda P., Diep L.M., Mwadime R., Kaaya A.N., Skaare A.B., Willumsen T., Westerberg A.C., Iversen P.O. (2018). Nutrition, hygiene, and stimulation education to improve growth, cognitive, language, and motor development among infants in Uganda: A cluster-randomized trial. Matern. Child Nutr..

[9] Yee A.Z., Lwin M.O., Ho S.S. (2017). The influence of parental practices on child promotive and preventive food consumption behaviors: A systematic review and meta-analysis. Int. J. Behav. Nutr. Phys. Act..

[10] Kristo A.S., Sikalidis A.K. (2014). Nutritional status and cardiometabolic risk factors among Turkish adolescent populations. Am. J. Biomed. Sci..

[11] Cuartas J., Jeong J., Rey-Guerra C., McCoy D.C., Yoshikawa H. (2020). Maternal, paternal, and other caregivers’ stimulation in low- and- middle-income countries. PLoS ONE.

[12] Aubel J. (2012). The role and influence of grandmothers on child nutrition: Culturally designated advisors and caregivers. Matern. Child Nutr..

[13] Martorell R., Zongrone A. (2012). Intergenerational influences on child growth and undernutrition. Paediatr. Perinat. Epidemiol..

[14] Frost M.B., Forste R., Haas D.W. (2005). Maternal education and child nutritional status in Bolivia: Finding the links. Soc. Sci. Med..

[15] Andriano L., Monden C.W.S. (2019). The Causal effect of maternal education on child mortality: Evidence from a quasi-experiment in Malawi and Uganda. Demography.

[16] Ickes S.B., Hurst T.E., Flax V.L. (2015). Maternal literacy, facility birth, and education are positively associated with better infant and young child feeding practices and nutritional status among Ugandan children. J. Nutr..

[17] Imdad A., Yakoob M.Y., Bhutta Z.A. (2011). Impact of maternal education about complementary feeding and provision of complementary foods on child growth in developing countries. BMC Public Health..

[18] Eshete H., Abebe Y., Loha E., Gebru T., Tesheme T. (2017). Nutritional Status and Effect of Maternal Employment among Children Aged 6-59 Months in Wolayta Sodo Town, Southern Ethiopia: A Cross-sectional Study. Ethiop. J. Health Sci..

[19] Bhargava A., Fox-Kean M. (2003). The effects of maternal education versus cognitive test scores on child nutrition in Kenya. Econ. Hum. Biol..

[20] Harding K.L., Aguayo V.M., Masters W.A., Webb P. (2018). Education and micronutrient deficiencies: An ecological study exploring interactions between women’s schooling and children’s micronutrient status. BMC Public Health..

[21] Rajna P.N., Mishra A.K., Krishnamoorthy S. (1998). Impact of maternal education and health services on child mortality in Uttar Pradesh, India. Asia Pac. Popul. J..

[22] Moestue H., Huttly S. (2008). Adult education and child nutrition: The role of family and community. J. Epidemiol. Community Health.

[23] Carlson G.J., Kordas K., Murray-Kolb L.E. (2015). Associations between women’s autonomy and child nutritional status: A review of the literature. Matern. Child Nutr..

[24] Nankinga O., Kwagala B., Walakira E.J. (2019). Maternal employment and child nutritional status in Uganda. PLoS ONE.

[25] Pratley P. (2016). Associations between quantitative measures of women’s empowerment and access to care and health status for mothers and their children: A systematic review of evidence from the developing world. Soc. Sci. Med..

[26] Mondal R.K., Majumder M.K., Rayhan S.J. (2014). The Impact of Maternal Education on Child Health; Evidence from Bangladesh. Asian J. Soc. Sci. Humanit..

[27] Karlsson O., De Neve J.W., Subramanian S.V. (2019). Weakening association of parental education: Analysis of child health outcomes in 43 low- and middle-income countries. Int. J. Epidemiol..

[28] Emina J.B., Kandala N.B., Inungu J., Ye Y. (2009). The Effect of Maternal Education on Child Nutritional Status in the Democratic Republic of Congo.

[29] Halder S., Kejriwal S. (2016). Nutritional Awareness of Mothers in Relation to Nutritional Status of the Preschool Children. Early Child Dev. Care.

[30] Chang A.Y., Cowling K., Micah A.E., Chapin A., Chen C.S., Ikilezi G., Sadat N., Tsakalos G., Wu J., Younker T. (2019). Past, present, and future of global health financing: A review of development assistance, government, out-of-pocket, and other private spending on health for 195 countries, 1995–2050. Lancet.

[31] Santas F., Celik Y., Eryurt M.A. (2018). Do health care reforms in Turkey have a significant effect in equal access to maternal and child health services in Turkey: An evidence from 20 years. Int. J. Health Plan. Manag..

[32] World Health Organization Publications on Turkey. https://www.euro.who.int/en/countries/turkey/publications.

[33] Sikalidis A.K., Öztağ M. (2020). Optimized snacking is positively associated with socioeconomic status and better Type 2 Diabetes Mellitus management in Turkish patients. Gazz. Med. Ital. Arch. Sci. Med..

[34] Huybrechts I., De Bacquer D., Matthys C., De Backer G., De Henauw S. (2006). Validity and reproducibility of a semi-quantitative food-frequency questionnaire for estimating calcium intake in Belgian preschool children. Br. J. Nutr..

[35] Turkish Ministry of Health. https://www.saglik.gov.tr/?_Dil=2.

[36] Neyzi O., Günöz H., Furman A., Bundak R., Gökçay G., Derendeliler F., Baş F. (2008). Body weight, height, head circumference and body mass index reference values in Turkish children. Child Health and Diseases.

[37] Bilgisayar Destekli Diyet ve Gida Programlari Beslenme Bilgi Sistemi. BeBis..

[38] Turkish Statistics Institute. https://www.tuik.gov.tr/.

[39] Koral S. (1991). Cultural, religious and socio-economic factors affecting sex education in Turkey. Plan. Parent. Eur..

[40] Arat Y. (2010). Religion, politics and gender equality in Turkey: Implications of a democratic paradox?. Third World Q..

[41] Shaheed F. (2010). Contested identities: Gendered politics, gendered religion in Pakistan. Third World Q..

[42] Cawley J., Liu F. (2012). Maternal employment and childhood obesity: A search for mechanisms in time use data. Econ. Hum. Biol..

[43] T.C. Başbakanlık Aile ve Sosyal Araştırmalar Genel Müdürlüğü (2010). Türkiye’de Aile Değerleri.

[44] Bianchi S.M. (2000). Maternal employment and time with children: Dramatic change or surprising continuity?. Demography.

[45] Economic Research Service (2004). Maternal Employment and Children’s Nutrition, Volume II, Other Nutrition-Related Outcomes.

[46] Unsar S., Dindar I., Kurt S. (2015). Activities of daily living, quality of life, social support and depression levels of elderly individuals in Turkish society. J. Pak. Med. Assoc..

[47] Oddo V.M., Mueller N.T., Pollack K.M., Surkan P.J., Bleich S.N., Jones-Smith J.C. (2017). Maternal employment and childhood overweight in low- and middle-income countries. Public Health Nutr..

[48] Anderson P.M., Butcher K.F., Levine P.B. (2003). Maternal employment and overweight children. J. Health Econ..

[49] Hawkins S.S., Cole T.J., Law C. (2008). Maternal employment and early childhood overweight: Findings from the UK Millennium Cohort Study. Int. J. Obes. (London).

[50] Morrissey T.W., Dunifon R.E., Kalil A. (2011). Maternal employment, work schedules, and children’s body mass index. Child Dev..

[51] Ziol-Guest K.M., Dunifon R.E., Kalil A. (2013). Parental employment and children’s body weight: Mothers, others, and mechanisms. Soc. Sci. Med..

[52] Taylor A.W., Winefield H., Kettler L., Roberts R., Gill T.K. (2012). A population study of 5 to 15 year olds: Full time maternal employment not associated with high BMI. The importance of screen-based activity, reading for pleasure and sleep duration in children’s BMI. Matern. Child Health J..

[53] Speirs K.E., Liechty J.M., Wu C.F., Strong Kids Research Team (2014). Sleep, but not other daily routines, mediates the association between maternal employment and BMI for preschool children. Sleep Med..

[54] Ystrom E., Barker M., Vollrath M.E. (2012). Impact of mothers’ negative affectivity, parental locus of control and child-feeding practices on dietary patterns of 3-year-old children: The MoBa Cohort Study. Matern. Child Nutr..

[55] Ystrom E., Niegel S., Vollrath M.E. (2009). The impact of maternal negative affectivity on dietary patterns of 18-month-old children in the Norwegian Mother and Child Cohort Study. Matern. Child Nutr..

[56] Akçil Ok M., Ercan A., Kaya F.S. (2016). A content analysis of food advertising on Turkish television. Health Promot. Int..

[57] Karaçam Z., Çoban A., Akbaş B., Karabulut E. (2018). Status of postpartum depression in Turkey: A meta-analysis. Health Care Women Int..

[58] Burchi F. (2010). Child nutrition in Mozambique in 2003: The role of mother’s schooling and nutrition knowledge. Econ. Hum. Biol..

[59] Yabancı N., Kısaç İ., Karakuş S.Ş. (2014). The Effects of Mother’s Nutritional Knowledge on Attitudes and Behaviors of Children about Nutrition. Procedia Soc. Behav. Sci..

[60] Alderman H., Behrman J.R., Glewwe P., Fernald L., Walker S., Bundy D.A.P., Silva N.D., Horton S., Jamison D.T., Patton G.C. (2017). Evidence of Impact of Interventions on Growth and Development during Early and Middle Childhood. Child and Adolescent Health and Development.

[61] Kosti R.I., Kanellopoulou A., Fragkedaki E., Notara V., Giannakopoulou S.P., Antonogeorgos G., Rojas-Gil A.P., Kornilaki E.N., Lagiou A., Panagiotakos D.B. (2020). The Influence of Adherence to the Mediterranean Diet among Children and Their Parents in Relation to Childhood Overweight/Obesity: A Cross-Sectional Study in Greece. Child Obes..

[62] Kanellopoulou A., Giannakopoulou S.P., Notara V., Antonogeorgos G., Rojas-Gil A.P., Kornilaki E.N., Konstantinou E., Lagiou A., Panagiotakos D.B. (2020). The association between adherence to the Mediterranean diet and childhood obesity; the role of family structure: Results from an epidemiological study in 1728 Greek students. Nutr. Health.

[63] Vanhelst J., Béghin L., Drumez E., Duhamel A., De Henauw S., Ruiz J.R., Kafatos A., Manios Y., Widhalm K., Mauro B. (2018). Adolescents’ diet quality in relation to their relatives’ and peers’ diet engagement and encouragement: The Healthy Lifestyle in Europe by Nutrition in Adolescence (HELENA) study. Public Health Nutr..

[64] Alper Z., Ercan İ., Uncu Y. (2018). A Meta-Analysis and an Evaluation of Trends in Obesity Prevalence among Children and Adolescents in Turkey: 1990 through 2015. J. Clin. Res. Pediatr. Endocrinol..

[65] Ozumut S.H., Erguven M., Besli E. (2020). Obesogenic Environment in Childhood: Implications of High Socioeconomic Level in a Developing Country. Medeni. Med. J..

[66] Turkish Ministry of National Education Publications Department (2005). Nutrition Education in Member States of the European Union and Recommendations for Turkey. J. Educ. Soc. Sci..

[67] Turkish Ministry of Health, Nutrition Section. https://hsgm.saglik.gov.tr/tr/beslenme/temel-besin-gruplari.html.

