# Supplementary material for: Traditional Societal Practices Can Avert Poor Dietary Habits and Reduce Obesity Risk in Preschool Children of Mothers with Low Socioeconomic Status and Unemployment
Source: Behav Sci (Basel). 2021 Mar 24;11(4):42. doi: 10.3390/bs11040042 (PMC8063956; doi:10.3390/bs11040042)
Supplement: Supplementary file 1 [file behavsci-11-00042-s001.pdf]

Supplementary materials

Table S1. Energy reference values for girls according to their physical activity levels.

Age (years)	Energy Requirement (resting) (kcal/day)	Total Energy Expenditure (kcal/day)		
		Low activity	Medium activity	High activity
4	821	1157	1321	N/A
5	868	1224	1398	1571
6	912	1286	1468	1651

Table S2. Energy reference values for boys according to their physical activity levels.

Age (years)	Energy Requirement (resting) (kcal/day)	Total Energy Expenditure (kcal/day)		
		Low activity	Medium activity	High activity
4	876	1235	1410	N/A
5	927	1307	1492	1677
6	979	1380	1576	1772

Table S3. Dietary reference values for protein intake.

Boys		Girls	
Age (years)	Dietary Reference Values (g/day)	Age (years)	Dietary Reference Values (g/day)
4	14	4	13.8
5	15.6	5	15.5
6	18.2	6	18

Table S4. Reference values for vitamin intake for both sexes age 4-6 years.	
Vitamin A (μg)	300
Vitamin C (mg)	30
Vitamin D (μg)	15
Vitamin E (mg)	9
Thiamine (mg)	0.6
Riboflavin (mg)	0.6
Niacin (mg)	6.7
Pantothenic acid (mg)	4
Pyridoxine (mg)	0.6
Biotin (μg)	25
B_{12} (μg)	1.5
Folate (μg)	140

Source: Turkish Ministry of Health, Turkey Dietary Guidelines, Ministry of Health of Turkey Publication No: 1046, Ankara 2016.
